# The Effect of a Silage Inoculant on Silage Quality, Aerobic Stability, and Meat Production on Farm Scale

**DOI:** 10.5402/2012/345927

**Published:** 2012-04-09

**Authors:** Y. Acosta Aragón, J. Jatkauskas, V. Vrotniakienė

**Affiliations:** ^1^Biomin Holding GmbH, Herzogenburg 3130, Austria; ^2^Department of Animal Nutrition and Feeds, Institute of Animal Science of Lithuanian Veterinary Academy, Baisogala 82317, Lithuania

## Abstract

The effect of inoculation on nutrient content, fermentation, aerobic stability, and beef cattle performance for whole-plant corn silage treated with a commercial product (blend of homo- and heterofermentative lactic acid bacteria, BSM, blend of *Enterococcus faecium*, *Lactobacillus plantarum*, and *Lactobacillus brevis*, DSM numbers 3530, 19457, and 23231, resp.), was compared to a control treatment with no silage additives (CT). The material had a DM of 323 g/kg, crude protein, and water-soluble carbohydrate concentrations of 87.9 and 110.5 g/kg DM, respectively. BSM increased the fermentation rate with a significantly deeper pH (*P* < 0.01), a significant increase in the total organic acids concentration (*P* < 0.05), more lactic acid (*P* < 0.01), and numerically more acetic acid compared to CT. BSM significantly decreased the concentrations of butyric acid (*P* < 0.01), ethanol, and ammonia-N compared to the CT. BSM-treated silage decreased DM by 3.0 % (*P* < 0.01) and had a higher digestible energy and a higher metabolizable energy concentration by 2.3 (*P* < 0.01) and 1.00 % (*P* < 0.05), respectively, compared to untreated silage. Aerobic stability improved by more than 2 days in BSM silage. The DM intake of silage treated with BSM increased by 6.14 %, and improved weight gain and the feed conversion by 8.0 (*P* < 0.01) and 3.4%.

## 1. Introduction

Today, silage is the world's largest fermentation process, with an estimated 287 million tons produced in EU alone [1]. For dairy and beef cattle farmers, their purpose is to produce more high-quality silage, rich in energy and protein.

The key factors influencing the feeding value of silages include crop characteristics, stage of crop development at ensiling, and the extent and type of fermentation achieved within the silo. Silage additives have gained more and more interest over recent years. It is widely accepted that silage additives can increase animal intake and animal performance through their effect on silage quality [2]. However, the market became reluctant to use acid additives as they were considered corrosive to machinery and concrete, and dangerous to those farm operatives who had to use them.

Successful silage production depends on the promotion of fermentation by beneficial bacteria [3], and therefore bacterial inoculants have been very popular, especially over the last 10 years.

Microbial inoculants have been added to silages to improve fermentation efficiency [4–6]. Microbial inoculants containing homofermentative lactic acid bacteria (LAB), in most of the cases *Lactobacillus plantarum*, are often added to silage because they very quickly produce large quantities of lactic acid, which lowers the pH of the silage [7, 8]. However, classic microbial inoculants often have no effect or can even make the aerobic stability of silages worse [7, 9, 10] because yeasts metabolize lactic acid to produce alcohol. Recently, the aerobic stability of a variety of silage crops has been markedly improved through inoculation with a heterolactic acid bacterium [11]. Improvements in aerobic stability brought about by this organism have been reported in corn silage [12]. Acetic acid produce from heterofermentative LAB inhibits the proliferation of yeasts in silage. Prevention of the growth of these organisms is crucial in restricting aerobic deterioration. A strain of heterofermentative LAB *Lactobacillus brevis *has been reported as a promising strain for improving the aerobic stability of silages [13].

The aim of this trial was to study the effect of a silage inoculant on the nutrient content, the silage quality, the aerobic stability, and the nutritive value of whole plant ensiled corn, as well as on the feed intake and growth performance of fattening young cattle.

## 2. Material and Methods

A trial was carried out with whole plant corn harvested at the milk/dough stage of maturity (32.3% DM, see Table 1) and used for ensiling, treated (BSM), or not (CT), with a silage inoculant.

The corn was chopped using a conventional forage harvester Massey Ferguson 5130 and ensiled directly after harvest in 2 horizontal silos with a capacity of 200 tones. The inoculant application rate of the commercial product was the rate recommended by the manufacturers (blend of *Enterococcus faecium*, *Lactobacillus plantarum*, and *Lactobacillus brevis*, DSM numbers 3530, 19457, and 23231 respectively; 4 g of product/ton of silage diluted in 4 l of water) to guarantee a concentration of 1 × 10^5^ cfu/g of material. The inoculant was applied uniformly using an applicator which was fixed on the harvester between the pick-up reel and cutting rollers. After weighing the untreated or inoculated chopped corn, it was transferred to one of two ferroconcrete trenches. Five control bags (made from four layers of cheesecloth) filled with 1 kg of ensiling mass were put in each silo to determine DM losses. The silos were filled within 48 hours and were covered with polythene sheet and weighted down with tires.

Five representative samples of harvested and chopped whole-plant corn were taken throughout the harvesting-ensilaging period. Silages were sampled every other week during the feeding experiment (14.12.2009 to 14.04.2010). At each sampling time two samples (approx. 500 g each) were taken 40–50 cm deep from the cut surface by coring vertically to the full depth of the silo using a 50 mm-silage corer.

Volatile fatty acid and lactic acid, as well as alcohol concentrations, were determined by gas-liquid chromatography on aqueous silage extracts obtained from steeping 30 g of fresh silage in 150 mL of deionized water for 16 hours at 4°C in a sealed container. This was followed by a preliminary filtering through 3 *μ*m filter paper. Deionized water (3 mL) from an internal standard solution (0.5 g 3-methyl-*n*-valeric acid in 1000 mL 0.15 mol/l oxalic acid) was added to 1 mL of filtrate from the above, and the solution was filtered through a 0.45 *μ*m polyethersulfone membrane into a chromatographic sample vial for analysis. A gas-liquid chromatograph SHIMADZU 2010 was used with a wide-bore capillary column (Stabilwax-DA 30 m, 0.53 mm, ID, 0.5 *μ*m) according to the Gas Chromatography and Biochemistry Analyzer official methods.

Aerobic stability was measured using data loggers which recorded temperature readings once every 6 hours from thermocouple wires placed in three replicate 1.500–2.000 g silage representative samples, which were aerated in open plastic bags and placed into open-top polystyrene boxes (volume about 3 liters). Thermocouple wires were inserted into the silage. The boxes were constantly kept at room temperature (21°C). Aerobic deterioration was denoted by days (or hours) until the start of a sustained increase in temperature by more than 2°C above the ambient temperature. 

For the animal feeding trial, 40 young beef cattle (8-9 months old) with similar mean live weight were used and divided into two analogous groups (20 animals each). The preexperimental adaptation for these animals lasted 21 days. The experimental period lasted 100 days.

During the preexperimental period, all animals were fed with untreated silage (CT) and a diet (CT + 4 kg of a commercial compound feed + 1 kg of barley straw) as during the experiment. During the experiment, each group was divided again into four subgroups, of five bulls each, and placed in four separate pens.

The animals were bedded on straw and had free access to water. Fresh silages were offered *ad libitum* twice daily, allowing for at least 10% orts (as-fed basis). Every 10 days the amount of silage fed and the refused silage were weighed over 2 consecutive days in order to calculate the daily silage intake. Silage DM intake was calculated per group as the difference between the amount of silage supplied and the amount of silage remaining. The compound feed was individually offered to all animals twice per day in a fixed amount. Barley straw was included in the diet (1 kg/animal/day; 88% of DM, energy value of 3.9 MJ ME/kg DM).

The animals were individually weighed on the first day of the experimental period and then once per month, and on the final day of the experiment. The average weight gain and growth rates were calculated for each animal and for each group. Feed conversion ratio was calculated as the ratio between feed intake and body weight gain.

Data was analyzed using variance analysis to test for the effect of silage treatments (Genstat, 1987). For the feed intake and feed conversion rates, a subgroup, of 5 beef cattle, was considered as the experimental unit. For body weight and daily weight gain, respectively, each animal within a group was considered an experimental unit. The Fisher's least significant difference (LSD) procedure at the 5% significance level was used to determine statistical differences between treatments. A probability of 0.05 < *P* < 0.10 was considered a near-significant trend.

## 3. Results and Discussion

The use of BSM significantly improved the silage quality compared to the CT (Table 2). The silage treated with BSM showed statistically significant higher DM recovery and digestible protein, coinciding with Merry et al. [14], lower DM losses (*P* < 0.01 for all) and higher crude protein content (*P* < 0.05). Its ADF content was significantly lower, and the metabolizable energy was higher (*P* < 0.05), whereas the digestible energy content was highly significant in the treated silage compared to the untreated silage. There were no significant differences between untreated and treated silages in crude fiber, NFE, WSC, and NDF content.

BSM treatment increased fermentation rates in whole-crop corn silages, resulting in a significant pH decrease (*P* < 0.01) and a significant increase in total organic acids concentration (*P* < 0.05) compared to the CT (Table 3). The lactic acid content in the BSM treatment was also significantly higher (*P* < 0.01) since homofermentative LAB were used [15]. The acetic acid content of the BSM treatment was numerically higher than that of the CT. Silage inoculation with BSM significantly decreased concentrations of butyric acid, ethanol, and ammonia-N (*P* < 0.01) of corn silage compared to the CT. Homofermentative silage inoculants by improving silage fermentation can reduce wasteful end products such as ammonia-N and volatile fatty acids, which result in poorer feed conversion efficiency and higher in-silo dry matter losses [16–18].

The use of silage inoculants containing homofermentative lactic acid bacteria, to increase lactic acid production and enhance the rate and extent of pH decline [19–21], can also lead to a reduction in protein breakdown [14]. As shown in Table 2, the BSM silage treatment decreased DM losses by 3.0% (*P* < 0.01) and had higher digestible energy (DE) and metabolic energy (ME) concentrations by 2.3 and 1.00% (*P* < 0.01 and *P* < 0.05), respectively, compared to the untreated CT silage. 

**Figure 1:**
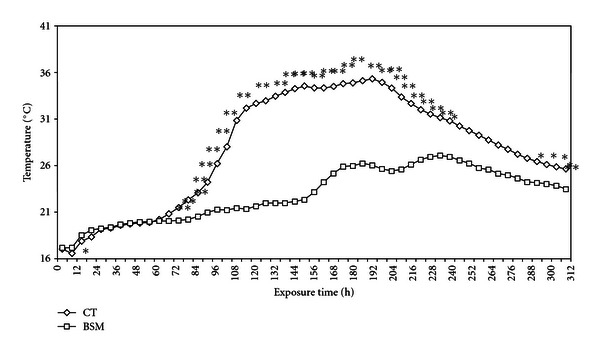
Temperature rise in a silage treated with a commercial product BSM (BSM) or not (CT). (Superscripts * and ** denote statistical differences of means at 0.05 and 0.01 levels, resp.).

During aerobic exposure after opening the silos, the CT (Figure 1)

- started to heat after 66 hours,
- had a temperature increase of more than 2°C above the ambient temperature after 84 hours, and
- reached a temperature of more than 14°C above the ambient temperature after 186 hours.

The temperature rise in the BSM treatment of more than 2°C above the ambient temperature occurred after 156 hours and reached a maximum temperature (+6°C above the ambient temperature) after 234 hours. 

Therefore, BSM silage was more stable by 72 hours (3 days) compared to the CT. Recently, silage studies with whole-crop corn silages using obligatory heterofermentative LAB *L. buchneri* as an inoculant showed a 20-fold increase in the aerobic stability of the silage, which increased from approximately 40 hours for untreated silages to more than 790 hours for the inoculated silages [22]. Other studies [12, 23] provide more definitive evidence for the existence of certain LAB strains with the power to inhibit yeast and molds growth, and to improve aerobic stability. Some authors have described the positive aspect of the formation of acetic acid by heterofermentative lactic acid bacteria, which inhibits spoilage organisms [24, 25]. 

No statistical differences were found between animals fed with BSM or CT silages at 0, 31, and 63 trial days in the live weight (Table 4). From the data presented it is obvious that, throughout the whole trial, animals fed with BSM silage achieved higher average weights compared to those from the CT. At the end of the experiment the difference in body weight reached 8.1 kg/animal, and this was considered a near-significant trend (0.05 < *P* < 0.10). 

Average daily weight gains (ADWGs) for BSM and CT are shown in Table 5. Between 0 and 31 trial days neither statistically, nor numerically marked, differences in ADWG were found between the treatments; however, in the trial period between 32 and 63 days the differences in ADWG show a near-significant trend (0.05 < *P* < 0.10) with a *P* value of 0.055. The ADWG in the last third of the feeding trial period (from 64 to 100 days), and throughout the whole trial period (0 to 100 days), showed a statistically significant difference (*P* < 0.01) of 138 and 80 g, respectively. 

In order to avoid differences due to different moisture contents, the intake is shown in Table 6 on the DM basis. Each mean is based on 4 observations. Randomized complete block where one pen is treated as a replication. 

The silage DM intake for BSM was higher by 6.14% compared to the CT (3.97 versus 3.74 kg DM/animal/day) and had a near-significant trend (*P* = 0.065). As expected, because of the restricted feeding, no differences were found in compound feed DM intake. These results were similar to those reported by Muck and Kung Jr. [7]; however, some researchers found that feeding microbial inoculated silage to cattle does not affect dry matter intake compared to noninoculated silage [26, 26]. A combination of increased DM intake and higher energy, in the silage treated with BSM, led to a significant increase (*P* < 0.05) in metabolizable energy intake compared to those animals fed with the CT. The animals receiving BSM had a better conversion of energy into body weight compared to that of the CT because they needed 2.37 MJ of ME (3.4%) less to increase the body weight by 1 kg. However, this difference was not statistically proven. 

## 4. Conclusions

The microbial silage inoculant which was tested, based on homo- and heterofermentative lactic bacteria, had a significant positive effect on whole-crop corn silage quality in terms of

- lowering pH and shifting fermentation towards lactic acid,
- suppressing butyric acid, ethanol, and ammonia-N formation,
- significantly reducing DM losses,
- statistically increasing digestible and metabolizable energy,
- statistical significant improvement of aerobic stability, and
- improvement of the silage intake and the performance of beef cattle, and a positive effect on the utilization of feed energy.

Therefore, it is concluded that using such a silage inoculant improves the whole-plant corn silage characteristics and the nutritive value for beef cattle. 

## Figures and Tables

**Table 1:** Chemical composition of whole plant corn at ensiling (*average of 5 samples*).

Parameter	Method	Units	Average	Standard deviation
Dry matter (DM)	Oven drying at 67°C for 24 h, equilibrated to room humidity overnight, milled through a 1 mm sieve and further dried at 105°C to constant weight.	g/kg	322.8	8.954
Crude protein	Kjeldahl-AOAC 984.13. 10.5 g of catalyst is used. With Block Digestion and Tecator Kjeltec system 1002 Distilling Unit	g/kg DM	87.9	2.124
Crude fat	Extraction by Soxtec System using petrol ether 40–600°C. Crude fat residue determined gravimetrically after drying		19.0	1.513
Crude fiber	With Fibercap (Foss Tecator) using sulphuric acid and Na hydroxide treatment		213.2	10.725
Nitrogen-free extract	Calculated		633.7	14.467
Crude ash	AOAC Method 942.05. Ca—AOAC 968.08 dry ashing, atomic absorption Spectrophotometry		46.2	3.850
Water-soluble carbohydrates (WSC)	Using the anthrone reaction assay (MAFF, 1986), from the herbage or silage extracts obtaining from steeping fresh herbage or silage in water		110.5	4.799
Digestible energy	Calculated	MJ/kg DM	12.95	0.051
Metabolizable energy	Calculated		10.69	0.102

**Table 2:** Effect of the treatment with a commercial product BSM on the chemical composition and fermentation characteristics of ensiled whole-plant corn.

Parameters	Unit	Treatments		Standard error	*P*
		Control *X* ± SD	BSM *X* ± SD		
Dry matter (DM)	g/kg	305.8 ± 4.30	312.2 ± 4.66	1.119	**
DM losses	g/kg DM	70.2 ± 15.87	40.9 ± 2.60	5.946	**
Crude protein		80.2 ± 4.94	84.7 ± 3.24	0.954	*
Digestible protein		48.2 ± 2.96	52.5 ± 2.01	0.680	**
Crude fat		19.7 ± 1.71	19.8 ± 1.70	0.341	0.845
Crude fiber		214.8 ± 4.59	210.2 ± 7.30	1.311	0.074
Nitrogen-free extract		640.1 ± 7.42	640.9 ± 11.38	1.919	0.833
Crude ash		45.2 ± 3.26	44.4 ± 4.10	0.744	0.622
Water-soluble carbohydrates (WSCs)		1.9 ± 1.36	2.1 ± 1.02	0.241	0.698
Neutral detergent fiber (NDF)		444.4 ± 11.73	439.1 ± 15.66	2.818	0.355
Acid detergent fiber (ADF)		238.3 ± 6.70	228.4 ± 12.24	2.221	*
Digestible energy (DE)	MJ/kg DM	12.8 ± 0.06	13.1 ± 0.07	0.031	**
Metabolizable energy (ME)		10.8 ± 0.08	10.9 ± 0.13	0.024	*

* and ** denote significance at level 0.05 and 0.01, respectively.

**Table 3:** Effect of the treatment with a commercial product BSM on the fermentation characteristics of ensiled corn.

Parameters	Unit	Treatments		Standard error	*P*
		Control *X* ± SD	BSM *X* ± SD		
pH	—	3.89 ± 0.09	3.71 ± 0.03	0.024	**
Total organic acids	g/kg DM	80.0 ± 4.33	93.3 ± 10.52	2.126	**
Lactic acid		50.3 ± 2.60	61.4 ± 5.88	1.472	**
Acetic acid		29.0 ± 2.16	31.5 ± 4.87	0.797	0.116
Butyric acid		0.4 ± 0.30	0.1 ± 0.11	0.055	**
Ethanol		13.2 ± 2.10	9.3 ± 2.41	0.606	**
Ammonia N	g/kg total N	51.0 ± 10.29	38.0 ± 7.77	2.271	**

* and ** denote significance at level 0.05 and 0.01, respectively.

**Table 4:** Average live weights of the beef cattle during the trial.

Treatment/statistical parameter	*n*	Trial day (kg, *X* ± SD)			
		0	31	63	100 (trial end)
Control	20	220.2 ± 11.83	249.1 ± 10.71	280.5 ± 9.80	320.0 ± 11.58
Commercial product BSM	20	220.3 ± 12.07	249.5 ± 12.81	283.5 ± 14.14	328.1 ± 14.55
Standard error	—	1.865	1.843	1.913	2.153
*P* level	—	0.973	0.912	0.440	0.058

**Table 5:** Average daily body weight gain of the beef cattle in different trial periods.

Treatment/statistical parameter	*n*	Trial period in days (kg, *X* ± SD)			
		0–31	32–63	64–100	0–100
Control	20	0.931 ± 0.124	0.981 ± 0.129	1.068 ± 0.074	0.998 ± 0.087
Commercial product BSM	20	0.940 ± 0.081	1.062 ± 0.129	1.206 ± 0.089	1.078 ± 0.078
Standard error	—	0.016	0.021	0.017	0.014
*P* level	—	0.778	0.055	**	**

**denotes significance at level 0.01.

**Table 6:** The effect of the treatment with the commercial product BSM on silage DM, energy intake, and feed conversion rate.

Parameter	Unit	Treatment		SE	*P*
		Control *X* ± SD	BSM *X* ± SD		
Silage DM intake	kg DM/animal/day	3.74 ± 0.12	3.97 ± 0.17	0.065	0.065
Compound feed DM intake		1.74 ± 0.0	1.74 ± 0.0	0.000	0.000
Total DM intake^1^		6.36 ± 0.12	6.59 ± 0.17	0.066	0.065
Total metabolizable energy (ME) intake	MJ/animal/day	69.27 ± 1.33	72.34 ± 1.97	0.799	*
Feed conversion rate	MJ of ME/kg gain	69.52 ± 3.49	67.15 ± 2.26	1.062	0.298

*denotes statistical significance at level 0.05.

^1^1 kg/animal/day of barley straw (88% of DM, 3.9 MJ ME/kg DM) was included in the diet for both treatments.
